# Highlights for the Management of a Child with Proteinuria and Hematuria

**DOI:** 10.1155/2012/768142

**Published:** 2012-07-12

**Authors:** Jyothsna Gattineni

**Affiliations:** Department of Pediatrics, University of Texas Southwestern Medical Center at Dallas, Dallas, TX 75235-9063, USA

## Abstract

The identification of hematuria or proteinuria in an otherwise healthy child can cause anxiety to both the family and the pediatrician. The etiology of hematuria and proteinuria includes a long list of conditions, and detailed workup can be exhaustive, expensive and not essential in most of the patients. As will be described in this paper, most of the children with proteinuria or hematuria have a benign etiology. The primary role of the pediatrician is to identify hematuria/proteinuria, recognize the common causes of hematuria/proteinuria, and more importantly identify children with serious conditions that need referral to the nephrologist in a timely manner.

## 1. Proteinuria

### 1.1. Introduction

The prevalence of isolated proteinuria detected by routine urinalysis (urine dipstick) in school age children was shown to be approximately 10% [1]. Further testing of these children revealed no evidence of significant renal disease in the absence of both hematuria and proteinuria. Similar findings were found in a study done on healthy adolescents [2]. Even though isolated proteinuria is usually benign, increased level of persistent proteinuria can be an indicator of progressive renal disease and is associated with increased cardiovascular morbidity [3–5]. Therefore, proteinuria presents a challenge to the primary care physician in regards to distinguishing benign proteinuria and proteinuria that requires workup and referral to the nephrologist. This section will discuss the different aspects of proteinuria including pathophysiology, etiology, and diagnostic workup of patients who present with proteinuria.

### 1.2. Pathophysiology

The glomerular filtration barrier provides the mechanical barrier between the blood and the urinary space. This barrier is comprised of the glomerular basement membrane, slit pores between the epithelial cell foot processes and the fenestrated endothelial cells. The glomerular filtration barrier is negatively charged due to the presence of glycosaminoglycans and glycocalyx [6]. Therefore, the nature of the particles that can cross this barrier is dependent not only on the molecular size of the particle but also on the charge of the particle. The vast majority of the proteins that are filtered by the filtration barrier are reabsorbed by the proximal tubule, and the remaining are degraded and excreted as low-molecular-weight proteins. About 30% of urinary proteins consist of albumin, transferrin, macroglobulin, and degraded filtered proteins. The remaining protein (70%) is the Tamm-Horsfall protein (secreted by the loop of Henle). Increased urinary protein losses can result from increased filtration across the filtration barrier (glomerular proteinuria), decreased reabsorption from the proximal tubule (tubular proteinuria) or increased secretion of protein from the tubules (secretory proteinuria).

### 1.3. Transient and Intermittent Proteinuria

Transient proteinuria is associated with fever, exercise, or stress and is not suggestive of underlying renal disease. When the underlying predisposing condition resolves, the proteinuria resolves. Another condition of intermittent proteinuria that causes concern for the parents and the pediatrician is orthostatic proteinuria. Orthostatic proteinuria is common in older children and adolescents with a prevalence of 2–5% [7]. Orthostatic proteinuria is the most common cause of proteinuria in adolescents (75%) [2]. The etiology is postulated as changes in glomerular hemodynamics due to postural changes, and orthostatic proteinuria rarely exceeds 1 gm/day. The first step in patients who present with persistent proteinuria is to do a spot urine protein creatinine ratio on a first morning urine specimen. Another option is to collect a split 24 hr urine collection based upon lying/supine position and upright position and not on the time of day.

### 1.4. Persistent Proteinuria

Persistent proteinuria (>4 mg/m^2^/hr of protein in a 24 hr urine collection or spot urine protein creatinine ratio of >0.2 mg/mg), as the name suggests is present on numerous occasions and needs to be evaluated further to rule out any underlying renal pathology. Glomerular causes for proteinuria are more common than tubulointerstitial causes for proteinuria, and the common causes are listed in Table 1 [8–10]. Of the glomerular causes, nephrotic syndrome is one of the important causes. Nephrotic syndrome is defined as protein excretion of >40 mg/m^2^/hr or >1 gm/m^2^/day in a 24 hr urine collection or a spot urine protein creatinine ratio of >2 mg/mg [10, 11]. Patients with nephrotic syndrome also have hypoalbuminemia, edema, and hyperlipidemia. Minimal change nephrotic syndrome is the most common histopathological diagnosis of nephrotic syndrome in children, and the typical age of presentation is 2–7 years and is more common in boys (2 : 1) [12]. Tubular proteinuria commonly consists of low-molecular-weight proteinuria. Dent's disease is an X-linked recessive disorder that presents with low molecular weight proteinuria, proximal tubulopathy, hypercalciuria, and nephrolithiasis. In the majority of patients with Dent's disease, there is an inactivating mutation of the CLCN5 gene (renal chloride channel). Lowes syndrome is also an X-linked disorder, and patients present with low molecular weight proteinuria, bilateral cataracts, proximal tubulopathy, and hypotonia. To diagnose tubular proteinuria, urinary studies looking for the excretion of low-molecular-weight proteins including *β*-2 microglobulin, retinol-binding protein, and *α*-1 microglobulin are necessary. Detailed discussion of these disorders is beyond the scope of this paper.

### 1.5. Approach to a Patient with Proteinuria

*History*. As with any medical problem, a thorough history is critical in evaluating a patient. History should include symptoms of swelling, headaches, hematuria, joint pains, rashes, elevated blood pressure, urinary tract infections, recent throat or skin infections, loss of appetite, decreased energy, weight loss, and intake of medications (please see Table 1 for examples). Family history is also important which should include cystic kidney disease, deafness, visual disturbances, or renal disease/renal failure/dialysis.

*Physical Examination. *Growth is an important clue for chronic diseases and needs to be measured. Blood pressure needs to be obtained and cross-referenced with normative published data [13]. Signs of flank pain, fluid overload, edema, organomegaly, rashes, joint swelling, anemia, and evidence of osteodystrophy should be examined. Please refer to Table 1 for the common conditions associated with persistent proteinuria to guide your physical exam for rare medical conditions.

*Laboratory Testing to Detect and Quantify Proteinuria*. Urinary dipsticks are commonly used to detect proteinuria and hematuria in the office setting, and they are good screening tools. The urine dipstick primarily detects albumin and does not detect low-molecular-weight proteins. This is due to the fact that albumin binds better to tetrabromophenol, which is the dye used in the dipstick. The color changes from yellow to green to blue with increasing amounts of protein in the urine, for example, trace (<20 mg/dl), 1+ (30 mg/dl), 2+ (100 mg/dl), 3+ (300 mg/dl), and 4+ (>2000 mg/dl) [14]. False negative results can be seen in very dilute urine samples especially when the specific gravity is <1.002 and with low molecular weight proteinuria. False positive results can be seen in highly concentrated urine samples, alkaline urine (pH > 8.0), after iodinated contrast, and with the use of antiseptics prior to urine collection. A quick way of quantifying proteinuria is measuring a spot/random urine protein creatinine ratio (mg/mg) when the urine dipstick shows persistent proteinuria (1+ and above). Many studies have shown a good correlation between spot urine protein creatinine ratio and 24 hr urinary protein excretion, and more importantly spot protein creatinine ratio can help the physician to decide which patients need further workup of proteinuria including a 24 hr urine collection [15–18]. The normal ratio for random urine protein creatinine ratio is <0.2, nephrotic range is >2 when both urine protein and creatinine are measured in mg/dl. When the spot protein creatinine ratio is between 0.2 and 2, it is advisable to obtain a 24 hr urine collection. Twenty-four-hour urine collection for protein quantification is the gold standard test, but it is inherent with many problems. Twenty-four-hour urine collections are not practical in children in diapers, and even if the child is toilet-trained, it is often associated with missed voids, inadequate collection, and volume errors. Normal protein excretion in children in 24 hr urine collection is <4 mg/m^2^/hr, nephrotic range proteinuria is >40 mg/m^2^/hr [14]. Abnormal proteinuria is from 4–40 mg/m^2^/hr on a 24 hr adequate urine collection.

*Laboratory Workup for Isolated Proteinuria*. It is paramount to establish if the proteinuria is transient, orthostatic, or persistent. In a patient who is asymptomatic with isolated proteinuria, urine dipstick needs to be repeated weekly on at least two occasions to establish that proteinuria was not transient. If the proteinuria disappears on repeat testing, then it is likely transient, and the family can be reassured. Urine dipsticks can then be repeated in 6 months–1 year [14]. In a patient with persistent proteinuria, to distinguish between orthostatic and persistent proteinuria, early morning spot protein creatinine ratio or split 24 hr urine collection should be obtained. While obtaining the split 24 hr urine collection, the most important aspect is that urine is collected based on lying/supine or upright position and not based on the timing of the day. Clear instructions need to be provided to the patients in regards to urine collection; one jug for while the patient is upright and one jug for after the patient has been supine for a considerable amount of time (overnight sleep). If the early morning urine protein creatinine ratio is <0.2 mg/mg or the protein excretion in the urine collected from lying/supine position is <60/m^2^/day, this is indicative of orthostatic proteinuria [9, 19]. Orthostatic proteinuria in longitudinal studies has shown favorable outcome without progression of renal disease [20]. If the urinary studies indicate persistent proteinuria, the patient needs a detailed and systematic workup including referral to a pediatric nephrologist. While the patient is waiting to be evaluated by a pediatric nephrologist, renal function tests (BUN and creatinine), albumin, and lipid profile can be obtained. Further evaluation will include a renal sonogram to rule out any structural malformations of the kidney, complement studies, and infectious workup based on the etiologies described in Table 1. The reader is advised to refer to the workup of proteinuria that is outlined in the publication by Hogg et al. [14].

## 2. Hematuria

### 2.1. Introduction

The incidence of macroscopic hematuria in children has been estimated to be 0.13% based on the data collected from 128,395 outpatient patient visits. In 56% of these patients, the cause was readily identifiable. In 26% of the children, the urine culture was positive, and only 9% had glomerular disease [21]. The incidence/prevalence of microscopic hematuria, which is more common than gross hematuria, varies in different studies due to the different criteria used to define microscopic hematuria. Using the definition of 10 or more red blood cells (RBCs) per high-power field (HPF) in two of the three consecutive urine samples, the point prevalence is 1-2% [22]. Using the criteria of 6 or more RBC/HPF in 4 or more urine samples, Vehaskari et al. showed the prevalence to be 0.37% [23]. The detection of hematuria results in immense anxiety for both the family and the pediatrician. In addition, detailed workup of every child with isolated hematuria results in a needless expense. However, it is important to identify children who could have serious underlying renal pathology. This section will discuss details about the pathophysiology, etiology, and workup of children who present with hematuria.

### 2.2. Overview and Pathophysiology

Hematuria is usually detected when the patient either presents with a change in their urine color, or when the urine is checked for other reasons. The urine dipsticks that are commonly employed to detect microscopic hematuria are very sensitive. When used correctly, urine dipsticks have a sensitivity of 100 and a specificity of 99 to detect 1–5 RBCs/HPF, which translates to 5–10 RBCs/*μ*l of urine [24, 25]. False positive results can be seen with hemoglobin, myoglobin, or hypochlorite in the urine [9]. Conversely, false negative results can be seen when the urine specific gravity is high or there are reducing agents like ascorbic acid in the urine [9]. The common causes of discolored urine are shown in Table 2. Therefore, a positive urine dipstick should be followed by urine microscopy to examine for red blood cells. Hematuria does not usually result in anemia [25]. Even 1ml of blood in 1000 ml of urine can change the urine color to red [26]. Red blood cells can arise from the glomeruli, renal tubules, interstitium, renal pelvis, ureter, bladder, or urethra. In children, glomerular hematuria is more common and is usually associated with RBC casts, deformed RBCs and/or proteinuria [25]. Ischemia of the renal papillae can be seen in sickle cell nephropathy and with certain medications/toxins. Hematuria is generally divided into two broad categories: macroscopic hematuria (visible to naked eye) and microscopic hematuria (not visible to naked eye). 

### 2.3. Macroscopic Hematuria

Macroscopic hematuria, as the name indicates, is visible to the naked eye. The first step in the evaluation of a patient with macroscopic hematuria is the color of the urine. Tea-colored, brown-colored or cola-colored urine is indicative of glomerular hematuria. The differential diagnosis includes postinfectious glomerulonephritis, membranoproliferative glomerulonephritis, rapidly progressive glomerulonephritis, IgA nephropathy, Henoch-Schönlein purpura, and hemolytic-uremic syndrome. The conditions mentioned above are usually associated with proteinuria and RBC casts and need prompt evaluation. In addition, some of the patients with these conditions can present with life-threatening hypertension or oliguria/anuria. Bright red-or pink-colored urine is indicative of bleeding from the urinary tract, past the glomerulus. The differential diagnosis includes tumor, trauma, hydronephrosis, renal calculus, cystitis, urinary tract infection, schistosomiasis (bilharziasis, Middle Eastern or African countries), tuberculosis of the urinary tract (endemic areas for tuberculosis), sickle cell trait, vascular anomalies, polyps, coagulopathy, renal artery or renal vein thrombosis, terminal hematuria (urethrorrhagia), or polycystic kidney disease [25, 26]. Nutcracker syndrome is another entity where the patient can present with intermittent gross or microscopic hematuria with orthostatic proteinuria. This phenomenon is due to compression of the left renal vein between the aorta and the superior mesenteric artery, which results in renal vein hypertension. Terminal hematuria (urethrorrhagia) can also result in gross hematuria (bright red color) or red staining of the undergarment. It is usually seen in prepubescent boys and can be associated with dysuria. Urethrorrhagia resolves spontaneously and does not need a detailed workup [29].

### 2.4. Microscopic Hematuria

As discussed earlier, there is no consensus on the definition of microscopic hematuria. In general, more than 5 RBCs/hpf is considered as microscopic hematuria. Patients with microscopic hematuria can be divided into two broad categories: asymptomatic isolated microscopic hematuria and symptomatic microscopic hematuria with positive family history and other associated features [25, 30].

In a large study done by Park et al. on school-aged children (7 million) in Korea, 1044 children had abnormal urinalysis. Of the 1044 children, isolated hematuria was found in 60% (719). Renal biopsy based on strict criteria (hypertension, severe proteinuria, family history of renal disease, abnormal renal function, or persistent hematuria and/or proteinuria for more than 12 months) was performed on a total of 113 children. Of the 719 children with isolated microscopic hematuria, 52 underwent a renal biopsy. Thirty-three children had thin basement membrane disease, 8 patients had IgA nephropathy, and 5 patients had membranoproliferative glomerulonephritis. As the criteria for performing a renal biopsy were stringent, the likelihood of finding significant renal disease was relatively higher in this group of children [31]. Another study was performed by Lee et al. where 461 renal biopsy cases were retrospectively analyzed [32]. The indications for renal biopsy in this study were isolated microscopic hematuria for 6 months or significant proteinuria (>2 g/24 hr urine) or the presence of both microscopic hematuria and proteinuria (>150 mg/24 hr urine). The biopsy criteria were less stringent than those in the study by Park et al. In the group with isolated microscopic hematuria (289 children), 136 (47%) children had no histopathological abnormality found on the renal biopsy. Thin basement membrane disease was found in 97 children (34%), and IgA nephropathy was found in 46 children (16%). The reason behind the increased number of normal renal biopsies in this study was due to the use of less stringent criteria for renal biopsy. These studies have demonstrated nicely that thin basement membrane disease is the most common cause of isolated microscopic hematuria, followed by IgA nephropathy, keeping in mind that almost half of the children with isolated microscopic hematuria might not have an identifiable cause. Thin basement membrane disease can be due to mutations in collagen IV and can be inherited in an autosomal dominant fashion. The long-term prognosis for thin basement membrane disease is favorable [33, 34]. IgA nephropathy can be progressive and needs close followup. End-stage renal disease can be seen in about 25% of pediatric patients with IgA nephropathy during a 20-year followup [35]. Treatment options for IgA nephropathy include angiotensin converting enzyme inhibitors/ angiotensin receptor blockers, immunosuppressive therapy, and fish oil supplements [35]. Hypercalciuria is another important differential for isolated microscopic hematuria. Based on different studies, hypercalciuria has been diagnosed in 10–30% of patients, who present with isolated microscopic hematuria [25, 30, 36, 37]. Dysfunctional voiding should also be considered during the evaluation of isolated microscopic hematuria. After reviewing the large population studies done on school children, it is safe to recommend that most of the children that present with isolated microscopic hematuria do not need an extensive workup at presentation, as they do not have significant underlying renal pathology [25, 30].

Another category is symptomatic microscopic hematuria, where the patient in addition to microscopic hematuria might have hypertension, proteinuria or family history of progressive renal disease, deafness or visual disturbances. This group will include patients with RBC casts, presence of proteinuria, symptoms suggestive of infectious processes, hypertension, or/and history of renal stones. The differential includes Alport syndrome, nephrocalcinosis, glomerulonephritis, or IgA nephropathy. These patients will need further evaluation including referral to a pediatric nephrologist.

### 2.5. Approach to a Patient with Hematuria

*History*. As always, obtaining a detailed history will guide the physician in the right direction and in a patient who presents with either gross or microscopic hematuria, the following questions will help formulate further workup and management. At first, it is important to ascertain the color of the urine as this will help distinguish between glomerular and nonglomerular hematuria. Bright red-colored urine usually indicates that the blood is coming from the ureter, bladder or urethra (non-glomerular). Glomerular hematuria in general is described as coca-cola-colored, tea-colored or dark-brown colored urine. However, if the urine has been in the bladder for a long period of time, even non-glomerular hematuria can present as brown colored-urine. The brown color is due to oxidation of the heme pigment [9]. Glomerular hematuria is usually painless. History of flank pain, radiating to the groin and dysuria, is suggestive of renal colic or nephrolithiasis. History of dysuria, fever with or without chills, suprapubic pain, flank pain, frequency of micturition, or recurrence of nocturnal enuresis is indicative of a urinary tract infection. History of sore throat 2-3 weeks prior to presentation or history of impetiginous rash 4–6 weeks prior to presentation is suggestive of postinfectious glomerulonephritis. Patients with Henoch-Schönlein purpura present with history of a purpuric/petechial rash, usually on the lower extremities (buttocks) but can be generalized and can also have associated symptoms of abdominal and/or joint pains. Patients with systemic lupus erythematosus present with history of facial rash across the nose and cheeks, joint pain, generalized malaise, or weight loss. Recurrent gross hematuria, especially soon after the onset of upper respiratory infection, is suggestive of IgA nephropathy or rarely thin basement membrane disease [33, 34, 38]. History of trauma, strenuous exercise, and menstruation: drugs and food history (food colorings, herbs, and toxins) should also be elicited. History of abdominal distension and or abdominal mass is suggestive of tumors, hydronephrosis (ureteropelvic junction obstruction), and polycystic or multicystic kidneys. In addition, history of child abuse should be considered and in adolescents, sexual activity should be inquired. The risk of urinary tract infections, cystitis, and urethritis is increased in sexually active teenagers.

Past medical history should include the presence of similar symptoms in the past, history of prior renal disease, and history of rashes or joint pains. Family history should include the presence of recurrent hematuria (thin basement membrane and nephrolithiasis), renal disease in relatives; history of deafness and chronic kidney disease/progressive renal disease is suggestive of Alport syndrome. History should also be ascertained about autosomal dominant polycystic kidney disease.

*Physical Examination*. As mentioned earlier, a thorough physical examination is important including measuring growth and vital signs. The presence of hypertension and edema in addition to hematuria is suggestive of acute nephritic syndromes, and thorough evaluation is essential. The absence of proteinuria and hypertension does not warrant immediate and thorough workup, but observation and followup is indicated. The presence of fever and loin pain is indicative of pyelonephritis. The presence of rashes or arthritis is indicative of systemic lupus erythematosus or Henoch-Schönlein nephritis. The palpation of an abdominal mass should raise suspicion for tumor, multicystic dysplastic kidney, polycystic kidney, or hydronephrosis.

*Workup of Hematuria*. *Gross Hematuria*. If the patient presents with gross hematuria, it is critical to examine the urine microscopically to confirm the presence of RBCs. If there are no RBCs, please refer to Table 2 to look for alternate causes of discolored urine. If the RBCs are present, the next step is to look for the origin of RBCs; examine for RBC casts or dysmorphic RBCs (phase contrast microscopy). A freshly voided urine sample is necessary to examine for RBC casts. RBC casts are usually not visible in a urine sample that has been in room temperature for a long time. In the presence of RBC casts, dysmorphic RBCs, proteinuria, hypertension, edema and oliguria, the hematuria is likely glomerular. The next step in the workup will include renal function (BUN and creatinine), electrolytes, albumin, complement studies (C3 and C4), and streptozyme test {antistreptolysin0 (ASO), antihyaluronidase (AH), anti-deoxyribonuclease B (anti-DNAse B), and antinicotinamide adenine dinucleotidase (anti-NADase)}, antinuclear antibody (ANA), and possibly antineutrophil cytoplasmic antibodies (ANCA). Urgent pediatric nephrology referral should be done in patients with RBC casts, dysmorphic RBCs, proteinuria, hypertension, edema, and oliguria. In the absence of dysmorphic RBCs, RBC casts and significant proteinuria, urological conditions and malignancies should be considered. All children who present with gross hematuria should have a renal ultrasound. If ultrasound reveals a structural abnormality or malignancy, urological referral is necessary. Spiral noncontrast CT scan is advised if nephrolithiasis is suspected. Cystoscopy is recommended when bladder pathology is suspected or to lateralize the location of the bleeding in a patient who has active recurrent hematuria. If the patient has fever, flank pain, and or dysuria, urine culture should be sent to rule out a urinary tract infection. 

*Microscopic Hematuria*. When the microscopic hematuria is isolated, asymptomatic and not associated proteinuria or hypertension, a step-wise and non-urgent workup is indicated. Repeat urine dipstick and microscopy can be repeated in 2-3 weeks. If microscopic hematuria resolves, no further workup is necessary. If isolated microscopic hematuria persists, spot urine calcium creatinine ratio and urinalysis on parents/siblings can be performed. The benefits of renal sonogram in this situation are not proven [30] but can relieve tremendous anxiety for the parents. If the above tests are normal, it is important to reassure the family that there are no life-threatening conditions, and the pediatrician can monitor the child with yearly urinalysis and blood pressure measurement. If the parents are insistent upon knowing the exact etiology of isolated microscopic hematuria, referral to a pediatric nephrologist should be considered. Patients with microscopic hematuria with symptoms or positive family history of renal disease and/or the presence of proteinuria warrant a referral to the pediatric nephrologist. Algorithms for workup of hematuria have been well described previously in, and the reader is advised to refer to the review articles [25, 26].

## 3. Summary 

As described in this paper, minority of patients who present with isolated microscopic hematuria or proteinuria have significant renal disease. This is based on mass urine screening studies on school-aged children. Basic screening tests can be employed to delineate transient urinary abnormalities from significant renal pathology. If the patient presents with proteinuria and hematuria, the likelihood of significant renal disease increases, and the pediatrician can initiate some of the workup as described above and refer the patient to a pediatric nephrologist. If the patient presents with hypertension, proteinuria, hematuria, and oliguria, an emergent referral to the pediatric nephrologist is recommended.

## Figures and Tables

**Table 1:** Causes of persistent proteinuria.

Persistent proteinuria	
Glomerular	Tubulointerstitial
**Diabetes**	**Acquired**
**Hypertension**	Acute tubular necrosis
**Reflux nephropathy**	Toxins (gold, lead, copper, and mercury)
**Primary glomerulonephropathy conditions**	Pyelonephritis
Minimal change nephrotic syndrome	Interstitial nephritis (penicillins and other antibiotics, NSAIDs, and penicillamine)
Focal and segmental glomerulosclerosis	**Inherited**
Membranous nephropathy	Proximal renal tubular acidosis
Membranoproliferative glomerulonephritis	Cystinosis
Congenital nephrotic syndrome	Galactosemia
**Secondary glomerulonephropathy conditions**	Lowe syndrome
IgA nephropathy	Dents disease
Infections (Hepatitis B and C, HIV, CMV, malaria, syphilis, streptococcal)	Wilson disease
Henoch-Schönlein nephritis and systemic lupus nephritis (SLE)	Tyrosinemia
Alport syndrome	
Thin basement membrane disease	
Hemolytic uremic syndrome	
***Malignancies***	
***Toxins***	

Adapted from [8, 9].

**Table 2:** Causes of discolored urine.

Red-colored urine	Hematuria (RBCs)
	Myoglobinuria (myoglobin and rhabdomyolysis)
	Hemoglobinuria (free hemoglobin)
	Porphyria (porphyrin)
	Urate crystals
	Foods (food coloring, beets, and blackberries)
	Drugs (phenolphthalein, chloroquine, phenazopyridine, iron sorbitol, desferrioxamine)

Dark yellow- or orange-colored urine	Concentrated urine
	Drugs (rifampin and pyridium)

Dark brown- or black-colored urine	Bile pigments
	Methemoglobinemia (methemoglobin)
	Melanin
	Alkaptonuria (homogentisic acid)

Adapted from [9, 25, 27, 28].
